# Evaluating the antioxidant and anti-inflammatory effect of melatonin in pediatric hemodialysis patients: a randomized, placebo-controlled trial

**DOI:** 10.1038/s41598-025-34264-0

**Published:** 2026-01-21

**Authors:** Ghadeer Amged Sayed, Radwa Maher El Borolossy, Ragia M. Said, Sara Mahmoud Shaheen

**Affiliations:** 1 Clinical Pharmacy Department, Faculty of Pharmacy, Ain Shams University, Cairo, Egypt; 2 Pediatric Nephrology Department, Faculty of Medicine, Ain Shams University, Cairo, Egypt

**Keywords:** Hemodialysis, Inflammation, Melatonin, Nephrology, Oxidative stress, Pediatrics, Diseases, Medical research, Nephrology

## Abstract

Children undergoing chronic hemodialysis are exposed to persistent oxidative stress and systemic inflammation, contributing to long-term cardiovascular complications. Melatonin (MLT) is a pleiotropic hormone with potential antioxidant and anti-inflammatory effects. Due to scarcity of studies on pediatrics this study sought to investigate the effects of MLT on oxidative stress and inflammation in pediatric hemodialysis patients. This prospective, block-randomized, double-blinded, placebo-controlled study aimed at assessing the effect of 5 mg MLT on oxidative stress and inflammation in pediatric hemodialysis patients. Forty eligible patients were randomly allocated into either MLT or placebo group. Serum malondialdehyde (MDA), nuclear factor kappa B (NF-κB) levels and lipid profile were measured at baseline and at the end of the study after 12 weeks. MLT significantly reduced the median percent change of serum NF-κB − 5.404(− 58.25–129.7) with p-value = 0.027 in addition to reduction in median total cholesterol in the MLT group from 163.7(134.5–259.5) at baseline to 144(113–242) at the end of the study with p-value = 0.038 and reduction of low-density lipoprotein levels from 96(78–183) to 78.5(48–171) at the end of the 12 weeks with p-value = 0.002 while there was no significance in the placebo group. Although, there was no statistical significance in serum MDA levels in the MLT group but significant increase in MDA levels in the placebo group was detected. MDA levels increased from 11.79 ± 5.078 to 14.79 ± 4.257 at the end of the study in the placebo group with p-value = 0.048, supplementation appears to have beneficial effects on ameliorating inflammation and reducing serum lipids. Moreover, MLT may have a protective antioxidant effect by reduction and inhibition of elevation of serum MDA levels.

*Trial registration*: The study was registered on ClinicalTrials.gov (identifier: NCT05570526 https://clinicaltrials.gov/study/NCT05570526), on 6th of October 2022

## Introduction

Chronic kidney disease (CKD) is one of the life-threatening conditions and a major health problem in the world. Despite being relatively uncommon in children, it can be a devastating illness with many long-term consequences. Moreover, disease etiology and cardiovascular complications in pediatric CKD influences the child health and also have a negative impact on their upcoming life^1^. In Egypt, CKD is probably the “tip of the iceberg,” where patients are diagnosed with renal disease when they have already reached the end-stage renal disease(ESRD)^2^

The progression of CKD to ESRD is attributed to enhanced oxidative stress and inflammation which exacerbate kidney dysfunction, causing accumulation of uremic toxins and cardiovascular complications^3,4^. Oxidative stress has been considered the link between inflammation and cardiovascular disease(CVD) in CKD^5^.

There is growing evidence that the risk of developing CVD in children with advanced CKD and ESRD is 30 times greater than that of age-matched controls. Therefore, children with CKD should also be considered at high risk for the development of CVD including endothelial dysfunction and atherosclerosis^6^.Many risk factors contribute to CVD in ESRD as dyslipidemia which is manifested by high triglycerides(TG),low density lipoprotein (LDL) and reduced high density lipoprotein levels (HDL) which predisposes them to atherosclerosis^7^.Not only dyslipidemia is the main contributor to endothelial dysfunction (ED) in these patients but also oxidative stress and inflammation plays pivotal role in this process^8,9^.Malondialdehyde (MDA) which is a product of lipid peroxidation and Nuclear factor kappa B (NF-κB ) which is key regulator of inflammation and immune response^10,11^are among the important key mediators in inflammation and oxidative stress process.

Melatonin (MLT) is a pleiotropic hormone secreted by the pineal gland. The main role of MLT is regulating circadian rhythm and transmitting information about sleep–wake cycles. Not only does MLT act as sleep regulator but also an effective antioxidant and anti-inflammatory. Moreover, MLT has a role in the detoxification of free radicals, bone formation and cardiovascular homeostasis^12–14^.It directly removes ROS (reactive oxygen species) products, including hydrogen peroxide and superoxide anions. It indirectly upregulates antioxidant enzymes such as: glutathione peroxidase (GPx), catalase (CAT), and superoxide dismutase (SOD)and reduces lipid peroxidation by decreasing MDA levels which acts as marker for oxidative stress in ESRD^15^.

Sleep-disturbances are more prevalent in CKD patient than other population specially those who are on maintenance hemodialysis (MHD)^16^. Nearly 80% of patients undergoing HD suffer from sleep problem^17^. Sleep disturbances in patients on MHD are caused both by renal disease and by the HD treatment itself. One of the important factors causing circadian rhythm disturbance is low levels of MLT in these patients^16^.

Clinical benefits of MLT in adult HD patients has been demonstrated in a randomized, double-blind, placebo-controlled trial, it showed that MLT supplementation could significantly regulate plasma levels of MDA, nitric oxide (NO), and total antioxidant capacity (TAC) compared to the placebo group in diabetic HD patients^18^. So, MLT may have potential promising reno protective effects in CKD patients receiving dialysis and improves the quality of life by enhancing sleep quality in pediatric HD patients.

So, our aim was to evaluate the efficacy and safety of MLT supplementation in pediatric patients undergoing MHD.

## Patients and methods

### Study design

The current study was block randomized, double blinded placebo-controlled study conducted at the pediatric hemodialysis unit of Ain Shams University hospitals, Cairo, Egypt from December 2022 to March 2023.

### Patient eligibility

All patients in the hemodialysis unit were assessed for eligibility. Inclusion criteria included male or female patients aged between 6 and 18 years old being on HD for at least 6 months prior to enrollment at a frequency more than or equal 3 times per week in a stable condition with no hospitalization during the previous 3 months.

While the exclusion criteria included patients taking any of the following: anti-epileptics, immunosuppressants, warfarin, or patients with some autoimmune conditions as systemic lupus erythematosus (SLE),rheumatoid arthritis (RA) or post-organ transplant, patients with malignancy or active inflammatory disease, patients with malabsorption, mental retardation or psychiatric illness, patients receiving any anti-oxidant during the past 3 months prior to participation, and participants in another clinical trial within the past 4 weeks or judged by the physician to be unsuitable.

**Fig. 1:**
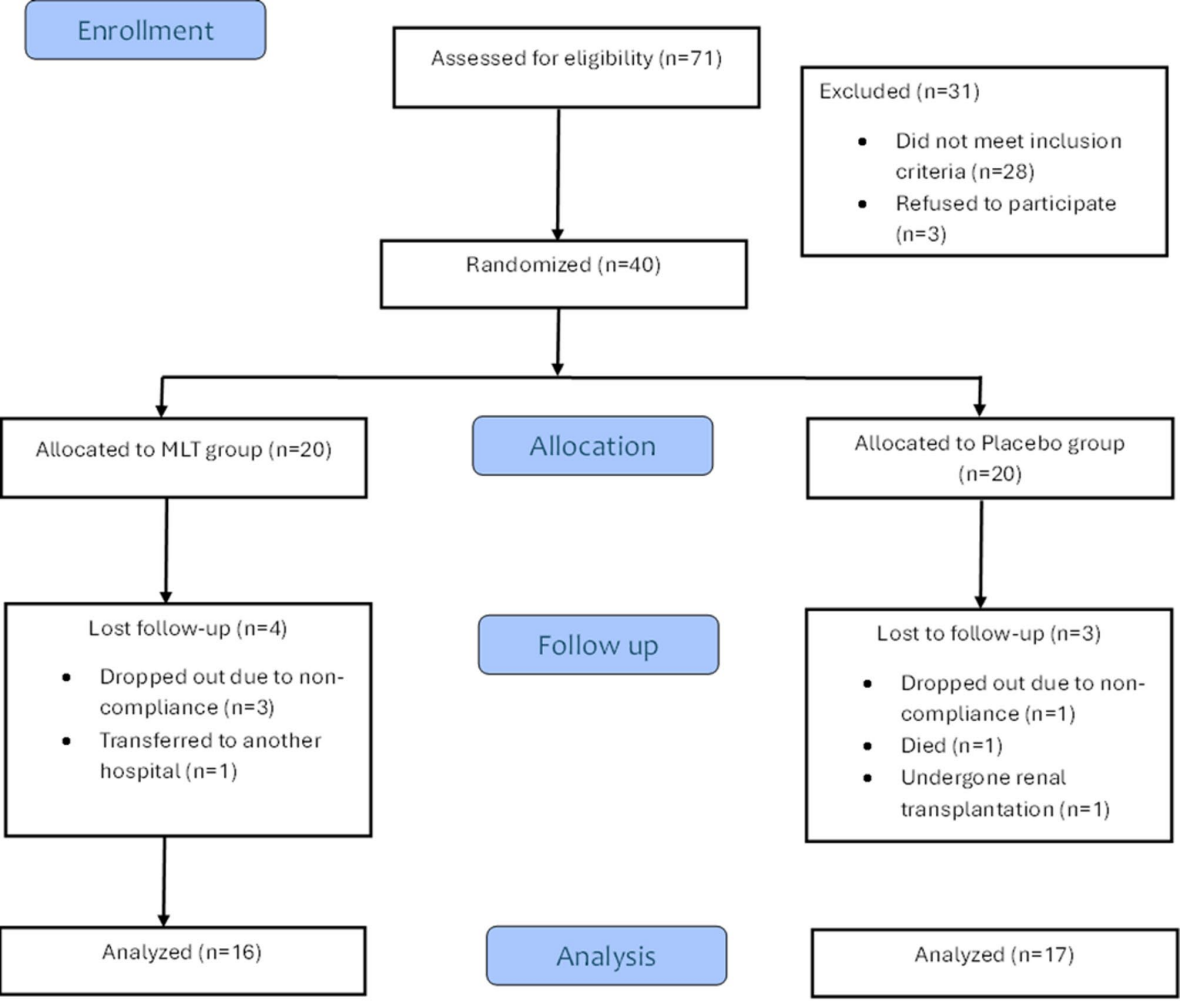
Summary of eligibility, enrolment and long-term follow-up for study procedures.

A total of (71) patients were assessed for eligibility; 31 patients were excluded (28 patients did not meet the inclusion criteria, and three patients declined to participate in the study) 0.40 patients were randomized into two groups, 20 patients in each as shown in Fig. 1

### Treatment intervention

Forty eligible patients were randomly allocated into either MLT or placebo group using 4-sided block randomization technique with randomization ratio (1:1). Within each block, possible allocation sequences included: AABB, BBAA, ABAB, BABA, ABBA, BAAB. A random number list generated with IBM SPSS Statistics, Version 20.0 (IBM Corp., Armonk, NY, USA) was used to assign one of the predefined block sequences, ensuring balanced allocation between groups. Neither the investigator (clinical pharmacist) nor the patients were aware of the study groups to ensure blinding. The medication and placebo were packed weekly in the containers by a clinical pharmacist and labelled by the patients’ name via one of the nurses and given to them weekly. The responsible physician was the only one who had access to the treatment allocation.

MLT group: 20 patients received 5mg of MLT tablets once daily 1-h before bedtime for 12 weeks supplied by Puritan’s Pride ®, USA in addition to their routine therapy which included vitamin D and calcium phosphate binders.

Placebo group: 20 patients received one tablet of placebo-starch based in similar pattern.

Placebo tablets were produced in similar shape, size and color by using pilot press tablet machine. All compounding, compression and testing done at Mash-Premiere pharmaceutical industries.

Either MLT or placebo tablets were placed weekly in containers to be supplied to the patients.

Patients were requested not to take any antioxidant or anti-inflammatory medication during the treatment period. To guarantee patients adherence to the medication or placebo, they were asked to return the empty containers or the remaining tablets weekly. Furthermore, parents of each participant were called every day via phone or sending messages to ensure that patients remember to administer their medication. Also, the patients were asked if they suffered from any side effects of the medication such as gastrointestinal symptoms including (nausea, vomiting and abdominal discomfort) or drowsiness or headache or skin rash. All patients enrolled in the trial were counselled about the disease and the management of dyslipidemia by decreasing saturated fats intake, processed foods and importance of exercise in order to decrease risks of CVD.

### Outcomes

Primary outcome: decrease in MDA level from baseline to week 12.

Secondary outcome: decrease in NF-κB level, decrease in total cholesterol, improvement of sleep quality which is demonstrated by decrease in global PSQI (Pittsburgh Sleep Quality Index) score and safety of MLT supplementation in this population.

### Hemodialysis protocol

All patients underwent conventional thrice weekly hemodialysis the session lasted for 3–4 h using high-flux biocompatible Fresenius polysulfone dialyzers with surface areas 0.7,1,1.4 m^2^ (Table 1). Dialysis efficiency was assessed by Kt/V, all patients achieved adequate dialysis efficiency with average Kt/V was 1.4 per session in MLT group and 1.43 in the placebo group throughout the study period. All patients’ access routes were arteriovenous fistula except for 1 patient in the placebo group was permcath.

### Clinical investigations and laboratory measurements

At baseline, both groups were subjected to history taking including age, gender, weight, height, HD duration, cause of CKD, comorbidities and medications taken.

Also, routine biochemical data were collected at baseline and after the end of the treatment phase of the study including complete blood count (CBC), urea, serum creatinine (SCr), sodium, potassium, calcium, phosphorous, albumin, alkaline phosphatase (ALP), parathyroid hormone (PTH) and eGFR was calculated using the creatinine-based “Bedside Schwartz” equation^19^

A 5-ml blood sample was withdrawn from each participant baseline and at the end of the 12 weeks just before their first weekly hemodialysis session on Saturday or Sunday early in the morning at 7:30 am after an overnight fast for the assay of parameters and routine laboratory tests. Samples were left for serum coagulation at room temperature for about 20 min then they were centrifuged at 3000 rpm for 20 min and stored frozen at -80°C.

Blood samples were withdrawn and assessed at baseline and at the end of the study using enzyme linked immunosorbent assay (ELISA) technique for determining MDA with a commercial kit “SunRed™ “ catalogue no.201–12-1372 (Hu Tai Road, Baoshan District, Shanghai, China.) and also NF-κB was determined by ELISA technique with a commercial kit “SunRed™ “ catalogue no.201–12-0691 (Hu Tai Road, Baoshan District, Shanghai, China.).Also lipid profile was assessed at baseline and at the end of the study, total cholesterol was measured by enzymatic colorimetric method (CHOD-PAP) using AGAPPE diagnostics kit catalogue no. 51403002 (Knonauerstrasse 54–6330 Cham-Switzerland),HDL-cholesterol was measured by precipitation of the reagent method (Mg acetate method) using AGAPPE diagnostics kit catalogue no. 51010001 (Knonauerstrasse 54–6330 Cham-Switzerland),Tri-glycerides were measured by enzymatic colorimetric method (GPO-PAP) method using AGAPPE diagnostics kit catalogue no. 5140004 (Knonauerstrasse 54–6330 Cham-Switzerland), LDL-cholesterol was calculated by Friedewald equation^20^.

In our study, Sleep quality was assessed by using PSQI using the Arabic validated version^21,22^ at baseline and at the end of 12-week MLT supplementation. This was done by asking the children each question in the questionnaire and their answer was documented by the researcher. Neither the researcher nor the patients knew which group they were assigned to ensure that there was no bias in the questionnaire results. PSQI contains 19 self-rated items that are combined in form of seven component scores, each of which has a range of 0–3 points. The seven component scores are then added to yield one global score with a range of 0–21 points of 7 components. The higher the score the poorer the quality of sleep^21^.

### Ethical considerations

This intervention was conducted in accordance with the principles outlined in the Good Clinical Practice standard and the Declaration of Helsinki.

The study protocol was revised and approved by the Committee of Ethics for experimental and clinical study at Faculty of Pharmacy Ain Shams University, Cairo, Egypt (RHDIRB2020110301 REC#99). Also, the study protocol is accessible through ClinicalTrials.gov where the study was registered (identifier: NCT05570526).

Prior to participation parents were informed about the study protocol and signed an informed consent statement on behalf of the children prior to inclusion in the study without any obligation to withdraw at any time if they want, per protocol analysis was performed.

### Sample size calculation

The sample size calculation was performed for the predicted changes in MDA levels. Based on previous data^23^the difference between MLT and placebo treated groups in MDA level after 12 weeks was 1.9 nmol/mL with pooled standard deviation 1.5 nmol/mL. Based on these findings, a minimal sample size of 17 subjects in each group is required at an alpha level of 0.05 and power of 95%. To compensate for loss to follow up, the sample will be increased by 15% to be 20 subjects in each group with total sample size of 40 subjects. Sample size was estimated using PS (Power and Sample Size Program) Version 3.1.2.

### Statistical analysis

Statistical analysis was performed using GraphPad prism version 10.1.2. The normality of data was determined by Kolmogorov–Smirnov test and Shapiro–Wilk test. Continuous variables were expressed as mean and standard deviation or median and interquartile range according to the distribution. Categorical (Qualitative) variables were expressed as frequencies and percentages. Parametric data statistical comparisons were done using paired and unpaired student t test, while non-parametric data were done using Mann Whitney U test and Wilcoxon rank test. Chi-square test and Fisher exact test were used to examine relationship between categorical variables. p-values < 0.05 were considered significant while p-values < 0.001 were considered highly significant.

## Results

A total of 71 patients on MHD were assessed for eligibility at pediatric hemodialysis unit Ain Shams university. Forty patients fulfilled the inclusion criteria, 33 patients completed the study ,6 patients lost to follow up, and 1 patient died in the placebo group the cause of death was unrelatedas summarized in figure 1.

**Baseline clinical characteristics and demographics** Baseline demographic data, clinical characteristics, comorbidities and medications were comparable in both MLT and placebo group except for hypertension as the number of hypertensive patients was higher in the MLT group and subsequently in the medications such as captopril and amlodipine there was significant difference (Table 1). No significant difference was detected between both groups in terms of baseline laboratory parameters (Table 2).

**Table 1:** Patient demographics and clinical characteristics.

Baseline evaluation	MLT group N = 16		Placebo group N = 17		p-value
A. Demographic data					
Gender					0.114^(a)^
Male	8(50%)		13(76.47%)		
Female	8(50%)		4(23.53%)		
Age	12.63 ± 2.156		10.88 ± 3.12		0.073^(b)^
BMI Z-score	− 1.117 ± 0.9939		− 0.9924 ± 1.785		0.807^(b)^
Hemodialysis duration (years)	2(1–5)		2(1–9)		0.614^(c)^
Average Kt/V	1.4		1.43		
B. Dialyzer material and surface area					
Fresenius polysulfone for all patients (33,100%)					
0.7m^2^	4(25%)		10(58.8%)		
1 m^2^	11(68.75%)		7(41.1%)		
1.4 m^2^	1(6.25%)		0(0%)		
C. Comorbidities					
Pericardial effusion	1(5.6%)		0(0%)		
Hypertension	11(68.75%)		4(23.53%)		0.009*^(a)^
Heart failure	3 (16.7%)		0(0%)		
D. Etiology					
Unknown	5(31.25%)		3(17.6%)		0.116^(d)^
GN	1(6.25%)		1(5.8%)		
Renal hypoplasia	6(37.5%)		4(23.5%)		
Hereditary nephritis	2(12.5%)		0		
Alport syndrome	1(6.25%)		0		
Reflux nephropathy	0		2(11.7%)		
Obstructive uropathy	1(6.25%)		1(5.8%)		
Nephronophthisis	0		4(23.5%)		
Polycystic kidney	0		2(11.76%)		
E. Medications					
Beta-blockers	4(25%)	1(5.88%)		0.174^(d)^	
Calcium phosphate binders	14(87.5%)	15(88.24%)		0.991^(d)^	
Vitamin D	15(93.75%)	17(100%)		0.484^(d)^	
Amlodipine	11(68.75%)	3(17.65%)		0.004*^(d)^	
Folic acid	11(68.75%)	11(64.71%)		0.805^(a)^	
Iron	4(25%)	1(5.88%)		0.174^(d)^	
Cinacalcet	2(12.5%)	5(29.41%)		0.398^(d)^	
Sevelamer	7(43.75%)	3(17.65%)		0.141^(d)^	
Captopril	7(43.75%)	1(5.88%)		0.016*^(d)^	
Doxazosin	6(37.5%)	2(11.76%)		0.117^(d)^	

All values presented as median(range) or n (%) or mean ± S.D. MLT: Melatonin, S.D: Standard deviation, n: number, BMI: Body mass index. (a)Chi square tests (b) T-test for equality of means (c) Mann Whittney test (d) Fisher exact test. *p-value < 0.05 is considered significant.

### Baseline laboratory evaluation and PSQI evaluation

At baseline, no significant difference was detected between both groups in terms of baseline laboratory parameters and in PSQI score (Table 2).

**Table 2:** Baseline laboratory measurements.

Baseline evaluation	MLT group N = 16	Placebo group N = 17	p-value
Hemoglobin (g/dL)	9.2 ± 1.325	9.459 ± 1.747	0.636^(a)^
WBCs (10^3^/μL)	7(2.3–8.5)	6(3.7–9.9)	0.782^(b)^
RBCs (10^6^/μL)	3.25 ± 0.4185	3.481 ± 0.6901	0.258^(a)^
HCT (%)	28.78 ± 3.952	30.28 ± 6.172	0.415^(a)^
MCV (fl)	88.87 ± 6.223	87.86 ± 6.343	0.649^(a)^
Platelets (10^3^/μL)	208(113–304)	263(117–621)	0.164^(b)^
Triglycerides (mg/dl)	143.3 ± 57.13	164.4 ± 51.27	0.272^(a)^
Total cholesterol (mg/dl)	163.7(134.5–259.5)	170(125–252.1)	0.527^(b)^
HDL (mg/dl)	32.83 ± 6.651	37.27 ± 9.118	0.121^(a)^
LDL (mg/dl)	96(78–183)	107(58–164)	0.824^(b)^
vLDL (mg/dl)	28.69 ± 11.46	32.94 ± 10.28	0.269^(a)^
TC/HDL	4.95(3.88–8.79)	4.52(2.97–9.52)	0.487^(b)^
LDL/HDL	3.12(2.12–6.29)	2.73(1.55–6.4)	0.387^(b)^
Urea (mg/dL)	154 ± 36.27	186.1 ± 77.52	0.141^(a)^
Serum Creatinine (mg/dL)	10.04 ± 1.506	8.741 ± 3.103	0.140^(a)^
Sodium (mmol/L)	137(126–140)	134(130–139)	0.122^(b)^
Potassium (mmol/L)	5.494 ± 1.004	5.406 ± 1.244	0.825^(a)^
Calcium (mg/dL)	8.813 ± 1.369	9.076 ± 1.103	0.545^(a)^
Phosphorus (mg/dL)	5.919 ± 1.501	6.118 ± 2.2	0.765^(a)^
Albumin (g/dL)	4.038 ± 0.4365	3.947 ± 0.4862	0.578^(a)^
ALP (U/L)	220(47–5799)	412(75–1155)	0.402^(b)^
PTH (pg/ml)	558.5(140–2251)	727(19–2659)	0.557^(b)^
eGFR (ml/min/1.73m^2^)	5.65(4.26–6.59)	5.68(4.16–7.27)	0.809^(b)^

All values represented as Mean ± S.D or median (range).MLT:Melatonin ,WBCs: White blood cells, RBCs: Red blood cells, HCT: Hematocrit, MCV: Mean corpuscular volume, TG: Triglycerides, TC: Total cholesterol, HDL: High density lipoprotein, LDL: Low density lipoprotein, vLDL: very low-density lipoprotein, ALP: Alkaline phosphatase, PTH: Parathyroid hormone, eGFR: estimated glomerular filtration rate.(a) T-test for equality of means (b) Mann Whittney test.

### End of study evaluation

#### Effect on MDA

After the three months treatment, levels of serum MDA in the placebo group had significantly increased after 12 weeks (14.79 ± 4.257) relative to baseline (11.79 ± 5.078) with p-value 0.048. Moreover, MDA levels in the MLT group decreased from (14.21 ± 8.041) at the beginning of the study to (13.29 ± 7.025). (Table 3).

Univariate analysis using ANCOVA was conducted to assess the effect of MLT treatment after adjusting for both baseline MDA and use of both Captopril and Amlodipine as antihypertensives, the model showed significant effect of baseline MDA (F = 6.752, p = 0.015, partial η^2^ = 0.189) indicating that baseline values were strong predictors of post-treatment MDA. The use of Captopril and Amlodipine showed non-significant effect (F = 1.450, p = 0.238, partial η^2^ = 0.048).

Also, the MLT group demonstrated marginal reduction in MDA compared to placebo group (F = 3.046, p = 0.092, partial η^2^ = 0.095), revealing a trend towards decreased oxidative stress that did not reach statistical significance.

#### Effect on NF-κB, Lipid profile

**Fig. 2:**
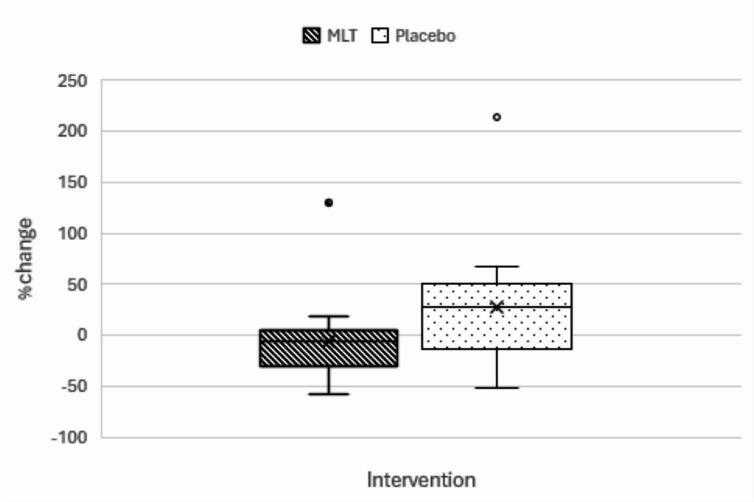
Boxplot representation of the percent change of NF-κB in both groups. Outlier, NF-κB : Nuclear factor kappa B.

After the three months treatment, MLT has succeeded in significantly reducing the percent change of NF-κB levels in MLT group (− 5.404% (-58.25–129.7)) relative to placebo (27.81% (− 51.28–213.6)) with p-value 0.027 as shown in Fig. 2. However, ANCOVA analysis adjusting for both baseline NF-κB and use of both Captopril and Amlodipine as antihypertensives revealed no significant reduction in NF-κB in MLT group relative to placebo group (F = 1.651, p = 0.209, partial η^2^ = 0.054), the model also showed significant effect of baseline NF-κB (F = 48.44, p < 0.001, partial η^2^ = 0.626) indicating that baseline values were strong predictors of post-treatment NF-κB . The use of Captopril and Amlodipine as antihypertensives showed non-significant effect (F = 0.039, p = 0.844, partial η^2^ = 0.001).This indicates that while the adjusted post treatment means were not significantly different when using baseline values as a covariate, the relative within subject reduction during treatment period was significantly higher in MLT group.

Also, MLT reduced total cholesterol significantly from 163.7(134.5–259.5) to 144 (113–242) with p-value 0.038 in MLT group. In addition there was a significant change in total cholesterol at the end of the study between the two groups with p-value 0.037.Serum LDL levels decreased significantly in MLT group from 96(78–183) at baseline to 78.5(48–171) at end of the study with p-value 0.002.Also,there was significant change in TC/HDL and LDL/HDL in MLT group with p-value 0.0105 and 0.0107 respectively.TC/HDL decreased from 4.95(3.88–8.79) at baseline to 3.87(2.23–6.91) at the end of the study while LDL/HDL decreased from 3.12(2.12–6.29) to 2.305(0.99–4.88). Moreover, there was no significant change in all other lipid profile parameters at the end of the study (Table 3).

**Table 3:** Baseline and end of study measurements for MDA, NF-κB , lipid profile and sleep quality index.

Parameter		MLT group N = 16	Placebo group N = 17	p-value
MDA (nmol/ml)	At baseline	14.21 ± 8.041	11.79 ± 5.078	0.307^(c)^
	After 12 weeks	13.29 ± 7.025	14.79 ± 4.257	0.459^(c)^
	**p-value**	0.637^(d)^	0.048* ^**(d)**^	
	% change	15.63 ± 66.58	46.02 ± 68.84	0.207^(c)^
NF-κB (ng/ml)	At baseline	4.657(2.13–17.34)	3.86(1.419–6.703)	0.191^(a)^
	After 12 weeks	4.169(2.217–12.36)	4.415(1.88–10.05)	0.845^(a)^
	**p-value**	0.104^(b)^	0.243^(b)^	
	% change	− 5.404(− 58.25–129.7)	27.81(− 51.28–213.6)	0.027* ^**(a)**^
PSQI	At baseline	4.5(3–7)	3(1–8)	0.063^(a)^
	After 12 weeks	2(1–6)	4(1–9)	0.122^(a)^
	**p-value**	0.0001** ^**(b)**^	0.582^(b)^	
	% change	− 41.667(− 83.33–0)	0(− 75–200)	0.002* ^**(a)**^
TG (mg/dl)	At baseline	143.3 ± 57.13	164.4 ± 51.27	0.272^(c)^
	After 12 weeks	126.9 ± 58.88	149.1 ± 45.83	0.234^(c)^
	**p-value**	0.062^(d)^	0.173^(d)^	
	%change	− 10.85 ± 24.24	− 6.207 ± 24.45	0.587^(c)^
TC (mg/dl)	At baseline	163.7(134.5–259.5)	170(125–252.1)	0.527^(a)^
	After 12 weeks	144(113–242)	157(138–230)	0.037* ^**(a)**^
	**p-value**	0.038* ^**(b)**^	0.686^(b)^	
	%change	− 11.99(− 51.08–26.04)	1.765(− 39.02–15.43)	0.344^(a)^
HDL (mg/dl)	At baseline	32.83 ± 6.651	37.27 ± 9.118	0.121^(c)^
	After 12 weeks	39.88 ± 12.87	38.88 ± 8.58	0.794^(c)^
	**p-value**	0.056^(d)^	0.361^(d)^	
	%change	24.65 ± 43.29	6.72 ± 21.64	0.138^(c)^
LDL (mg/dl)	At baseline	96(78–183)	107(58–164)	0.824^(a)^
	After 12 weeks	78.5(48–171)	87(66–162)	0.119^(a)^
	**p-value**	0.002* ^**(b)**^	0.229^(b)^	
	%change	− 18.8 ± 24.15	− 6.806 ± 27.64	0.195^(c)^
vLDL (mg/dl)	At baseline	28.69 ± 11.46	32.94 ± 10.28	0.269^(c)^
	After 12 weeks	25.38 ± 11.85	30.18 ± 9.268	0.202^(c)^
	**p-value**	0.056^(d)^	0.241^(d)^	
	%change	− 11.02 ± 23.67	− 4.847 ± 27.56	0.496^(d)^
TC/HDL	At baseline	4.95(3.88–8.79)	4.52(2.97–9.52)	0.487^(a)^
	After 12 weeks	3.87(2.23–6.91)	4.29(3.11–7.93)	0.539^(a)^
	**p-value**	0.0105* ^**(b)**^	0.159^(b)^	
	%change	− 20.69 ± 26.2	− 8.41 ± 25.01	0.178^(c)^
LDL/HDL	At baseline	3.12(2.12–6.29)	2.73(1.55–6.4)	0.387^(a)^
	After 12 weeks	2.305(0.99–4.88)	2.505(1.6–5.42)	0.539^(a)^
	**p-value**	0.0107* ^**(b)**^	0.211^(b)^	
	%change	− 25.69 ± 34.69	− 7.035 ± 37.58	0.149^(c)^

All values represented as mean ± S.D or median(range). MLT: Melatonin, S.D: Standard deviation, MDA: Malon dialdehyde, NF-κB : Nuclear factor kappa B, PSQI: Pittsburgh Sleep Quality Index, TG: Triglycerides, TC: Total cholesterol, HDL: High density lipoprotein, LDL: Low density lipoprotein, vLDL: very low-density lipoprotein. (a) Mann Whittney test (b) Wilcoxon matched pairs signed rank test (c) T-test for equality of means (d) Paired t-test. * p-value < 0.05 is considered significant **p-value < 0.001 is considered highly significant.

#### Effect on PSQI

**Fig. 3:**
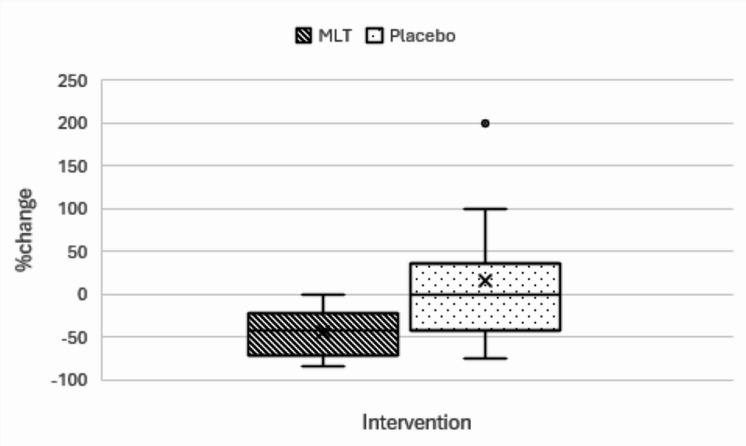
Boxplot representation of the percent change in PSQI global score in both groups. Outlier, PSQI: Pittsburgh Sleep Quality Index.

In addition to MLT’s benefits to ameliorate inflammation and oxidative stress, there was also highly significant change in the PSQI value in MLT group before and after treatment with p-value 0.0001 also the percent change in the PSQI score between both groups is significant − 41.667%(-83.33–0)in MLT group and 0%(− 75–200) in placebo group with p-value 0.002.So,This means that MLT supplementation can enhance the quality of sleep in this population and decrease sleep irregularities as shown in Fig. 3.

#### Drug safety

MLT was well tolerated without any reported side effects except for somnolence which was desired to enhance the sleep quality of those patients, and the drug was taken before bedtime, so it was not considered a problem. Only one patient reported suffering from abdominal pain in the MLT group and another one in the placebo group suffered from headache. This was not tested as no p-value could be obtained due to small number of cases within subgroups. Also, other safety parameters were measured as serum electrolytes, CBC ,serum creatinine, urea, PTH ,albumin and ALP nothing was altered except for total leucocytic count increased significantly but still within the normal range in pediatric population which is (4500–13,500 /μL)^24^ (Table 4).

**Table 4:** Baseline and end of study measurements for routine biochemical data and safety parameters.

Parameter		MLT group N = 16	Placebo group N = 17	p-value
Hb (g/dL)	At baseline	9.2 ± 1.325	9.459 ± 1.747	0.636^(a)^
	After 12 weeks	9.581 ± 0.6014	9.747 ± 2.037	0.756^(a)^
	p-value	0.347^(b)^	0.613^(b)^	
RBCs (10^6^/μL)	At baseline	3.25 ± 0.4185	3.481 ± 0.6901	0.258^(a)^
	After 12 weeks	3.384 ± 0.4006	3.67 ± 0.9032	0.254^(a)^
	p-value	0.3303^(b)^	0.387^(b)^	
WBCs (10^3^/μL)	At baseline	7(2.3–8.5)	6(3.7–9.9)	0.782^(c)^
	After 12 weeks	5.8(2.9–7.2)	7.1(3.5–11.8)	0.019*^(c)^
	p-value	0.1301^(d)^	0.055^(d)^	
Platelets (10^3^/μL)	At baseline	208(113–304)	263(117–621)	0.164^(c)^
	After 12 weeks	198.5(85–344)	222(31–414)	0.470^(c)^
	p-value	0.733^(d)^	0.148^(d)^	
Sodium (mmol/L)	At baseline	137(126–140)	134(130–139)	0.122^(c)^
	After 12 weeks	135.5(128–146)	134(130–139)	0.184^(c)^
	p-value	0.826^(d)^	0.861^(d)^	
Potassium (mmol/L)	At baseline	5.494 ± 1.004	5.406 ± 1.244	0.825^(a)^
	After 12 weeks	5.306 ± 0.8054	4.982 ± 1.192	0.370^(a)^
	p-value	0.453^(b)^	0.096^(b)^	
Calcium (mg/dL)	At baseline	8.813 ± 1.369	9.076 ± 1.103	0.545^(a)^
	After 12 weeks	8.763 ± 1.346	8.812 ± 1.095	0.908^(a)^
	p-value	0.883^(b)^	0.271^(b)^	
Phosphorus (mg/dL)	At baseline	5.919 ± 1.501	6.118 ± 2.2	0.765 ^(a)^
	After 12 weeks	5.9 ± 1.956	5.435 ± 2.851	0.591 ^(a)^
	p-value	0.972^(b)^	0.148^(b)^	

All values represented as mean ± S.D or median(range)).MLT: Melatonin, Hb: Hemoglobin, WBCs: White blood cells, RBCs: Red blood cells, HCT: Hematocrit, MCV: Mean corpuscular volume, ALP: Alkaline phosphatase, PTH: Parathyroid hormone, eGFR: estimated glomerular filtration rate.(a) T-test for equality of means (b) Paired t-test (c) Mann Whittney test (d) Wilcoxon matched-pairs signed rank test. *p-value﻿<0.05 is considered significant

## Discussion

Oxidative stress and inflammation play a crucial role in the pathophysiology of CKD, particularly in patients undergoing MHD. They are the main mediators of the progression of CKD and its cardiovascular complications^3^. MDA is a well-established biomarker of lipid peroxidation and oxidative stress^11^ and NF-κB is key regulator of inflammation and immune response ^10^ both are among the important key mediators in inflammation and oxidative stress process. MLT acts as an effective antioxidant that directly removes ROS products and indirectly upregulates antioxidant enzymes and reduces lipid peroxidation by decreasing MDA levels^25^.

To the best of our knowledge this is the first randomized controlled trial to investigate the effect of MLT on oxidative stress and inflammation pediatric HD patients.

According to the results found in the current study, after 12 weeks of MLT supplementation in children undergoing MHD we noticed reduction in serum MDA levels in MLT group from 14.21 ± 8.041 nmol/ml at baseline to 13.29 ± 7.025 nmol/ml at the end of our trial. But unfortunately, this reduction did not achieve statistical significance. Fortunately, the level of serum MDA in the placebo group showed a statistically significant increase in the mean value from 11.79 ± 5.078 nmol/ml at baseline to 14.79 ± 4.257 nmol/ml at the end of the study with p-value 0.048.

Also, In the current study, when using ANCOVA model with adjusting for baseline MDA levels and the use of Captopril and Amlodipine as antihypertensive medications, MLT demonstrated a trend toward reducing oxidative stress, although the reduction did not reach statistical significance. The strong predictive effect of baseline MDA underscores the importance of initial oxidative status in determining treatment response. This suggests that patients with higher baseline MDA may require longer treatment duration to achieve significant reduction. The lack of effect of Captopril and Amlodipine on post-treatment MDA indicates that the observed changes are likely related to MLT itself rather than the use of anti-hypertensives. Although there is no statistical significance in the MLT group, this result still has clinical relevance confirming that MLT has a protective antioxidant effect by reduction and inhibition of elevation of serum MDA levels (oxidative stress marker) in pediatric HD patients.

Due to the limited number of studies investigating the effects of MLT on oxidative stress and inflammation in children, research in this area remains scarce so, our findings were compared to those in adults, where similar results were observed in a clinical trial by Sadeghi et al.^26^ on diabetic CKD adult patients administrating 10 mg of MLT daily for 10 weeks who found that MLT supplementation did not significantly change oxidative stress levels and inflammatory markers including MDA, IL-6 (Interleukin-6),TAC and CRP(C-reactive protein).Sadeghi and its colleagues reported that mean MDA value at the beginning of the trial was 14.3 ± 8 in the MLT group versus 12.8 ± 2.8 in the placebo group by the end of their trial MDA decreased to 14.1 ± 5.3 in MLT group while in the control group it decreased also to 12.3 ± 2.9 with p-value 0.22, which was different from our findings regarding the MDA level in the placebo group which was increased significantly agreeing the fact that MLT has protective effect against oxidative stress.

Also, Kücükakin et al.^27^ showed that MLT had no effect on oxidative stress marker MDA in patients undergoing major vascular surgery, where it failed to modify surgical stress response.

However, in a study performed by Ostadmohammadi et al. ^18^ using 5mg MLT twice daily in diabetic HD patients, the results demonstrated MLT’s ability to ameliorate oxidative stress and inflammation by significantly reducing the level of serum MDA, increasing total antioxidant capacity and also decreasing CRP level. In their study MLT reduced MDA value (β = − 0.21 μmol/L ,95% CI − 0.36 to− 0.06, P = 0.005).

Our results contrast Mohammed et al.^28^ findings which used 1 mg MLT for three months in pediatric autistic patients resulted in the decrease of MDA level from 5.46 ± 2.67 at baseline to 3.08 ± 1.46 at the end of treatment with p-value < 0.001.

The reasons that our results didn’t reach significant reduction in MDA levels after 3 months of MLT administration may be due to the large number or dropouts which were nondrug related.

Since, inflammation is a strong indicator of poor consequences in patients undergoing long-term MHD^29^.NF-κB is the key regulator of production of pro-inflammatory cytokines (TNF-α,IL-6) and its activation contributes to the inflammatory burden as seen in these patients^30^.

This is the first study to explore the effect of MLT on NF-κB in this population and due to lack of studies on pediatrics. Our results were in accordance with those found in in vivo studies on rat models^31^ which demonstrated that MLT supplementation resulted in the reduction of NF-κB and MDA levels in rats with renal mass reduction, while the results of the current study found significant reduction of serum NF-κB in MLT group with median percent change − 5.404% (-58.25–129.7) versus increase in placebo group 27.81% (− 51.28–213.6) with p-value 0.027 which is considered statistically significant. Our findings confirm the results of previous pre-clinical studies and ensure the promising effect of MLT on inflammatory marker NF-κB.

However, ANCOVA model did not show a statistically significant difference in post-treatment NF-κB levels between groups after adjusting for baseline values, the MLT group demonstrated greater relative within group reduction during the treatment period. This suggests the potential anti-inflammatory effect of MLT that may not have been fully detected when baseline values were controlled for. The strong influence of baseline NF-κB values on post treatment levels likely reduced the statistical power between group analysis. Nonetheless, the direction and magnitude of the within group reduction supports the hypothesis that MLT may reduce inflammation.

One of the major problems that HD patients suffer from is sleep disturbances which is more prevalent in patients with CKD, ESRD and dialysis populations than the general population^32^. Approximately 80% of patients undergoing MHD suffer from sleep problems^17^. Studies have proven that sleep problems in patients on dialysis have a major negative impact on their quality of life and increases morbidity^33^. Sleep disturbances in patients who are on MHD are caused both by renal disease and by the HD treatment itself. Moreover, in ESRD and dialysis, sleep/wake cycles and circadian rhythmicity may be affected due to disturbing MLT levels^34^

In this study, compared to placebo, the median PSQI score in the MLT group decreased significantly from 4.5(3–7) at baseline to 2(1–6) at the end of our trial with p-value 0.0001 which is highly significant. On the other hand, in the placebo group the median PSQI score increased from 3(1–8) at baseline to 4(1–9) after 12 weeks. This indicates that MLT effectively enhances the sleep quality and reduces sleep disorders in pediatric hemodialysis patients.

Our results align with El Shahat et al.^35^ in their study where they used exogenous MLT to enhance sleep quality in HD patients. They found a decrease in global PSQI score in the MLT group compared to placebo with p-value < 0.001.

It is well established that ESRD patients on MHD suffer from reduction in HDL cholesterol levels and increase in serum triglycerides and total cholesterol levels which in turn lead to atherosclerosis and CVD which is prevalent widely in this population^7^.

In this clinical trial, we noticed the reduction of total cholesterol and LDL in both groups. This is due to patient counselling about the importance of lifestyle modification in their diet such as decreasing saturated fats intake, processed foods and importance of exercise in order to decrease risks of CVD. Nevertheless, significant reduction (p-value 0.002) in LDL and TC (p-value 0.038) was noticed in the MLT group indicating the promising effect of MLT on reduction of serum lipids. Despite that the increase in HDL in the MLT group was not significant but this increase was more than that in the placebo group.

Additionally, our study showed decrease in TC/HDL and LDL/HDL significantly only in the intervention group (p-value 0.01). Since, TC/HDL ratio is a strong predictor of coronary heart disease and considered more sensitive than total cholesterol alone, as it better describes the presence of atherogenic non-HDL particles in comparison to non-atherogenic HDL particles^36^.So, this indicates MLT’s ability to decrease atherosclerosis risk and protect against CVD.

Zahed et al.^37^ reported in their study that MLT supplementation in HD patients with 3 mg/day at bedtime for 12 weeks improved serum TG and HDL levels in but had no effect on TC.

Previous meta-analysis showed that triglycerides levels were significantly decreased only at doses ≥ 8 mg/ day and when the trial lasted for ≥ 8 weeks. Total cholesterol levels were also significantly reduced only at doses 8 mg/day and when baseline total cholesterol levels were equal or higher than 200 mg/dL but did not influence other lipid profile parameters^38^.

Effects of MLT on lipid profile in this study was in contrast to Ostadmohammadi et al. findings ^18^which demonstrated that the use of 10 mg MLT daily for 12 weeks in diabetic HD patients did not affect lipid profile.

## Conclusion

The administration of 5 mg MLT daily reduced NF-κB, TC, LDL and enhanced sleep quality in ESRD patients on MHD. Our study revealed that MLT is relatively safe and not associated with any significant adverse effects in these patients. To summarize, MLT is considered effective supplement that mitigates inflammation and has protective effect against oxidative stress, so it is considered protective against CVD.

### Limitations

There are few limitations in the current study, the duration of MLT treatment may be increased in future research in order to assess whether longer duration affects more on MDA and NF-κB , Also the large number of dropouts which was nondrug related and that we did not asses serum MLT level which may add clinical relevance in order to see the difference between before and after administration of MLT.

### Recommendations

Larger controlled trials are needed to confirm MLT’s efficacy on larger population, using higher dose with longer duration, and measure more markers for oxidative stress and inflammation.

Though, large scale studies with larger sample size, higher dose and duration are recommended in order to affirm the efficacy of long-term MLT supplementation in pediatric HD patients Also, measuring more oxidative stress markers as SOD or GPx and inflammatory markers as TNF-α (Tumor necrosis factor alpha) or IL-6 (Interleukin 6) may add clinical relevance.

## Data Availability

The datasets and the statistical code are available only upon justified scientific request due to patient confidentiality. Any requests should be addressed to the corresponding author.
